# *SPORTS STARS* study protocol: a randomised, controlled trial of the effectiveness of a physiotherapist-led modified sport intervention for ambulant school-aged children with cerebral palsy

**DOI:** 10.1186/s12887-018-1190-z

**Published:** 2018-08-02

**Authors:** Georgina L. Clutterbuck, Megan L. Auld, Leanne M. Johnston

**Affiliations:** 1 The University of Queensland, School of Health & Rehabilitation Sciences, Brisbane, Australia; 2 The Cerebral Palsy League, Brisbane, Australia

**Keywords:** Cerebral palsy, Physiotherapy, Sport, Modified sport, Gross motor, Exercise, Group, School aged

## Abstract

**Background:**

Modified sport interventions run by physiotherapists have shown potential as cost-effective, engaging, and effective interventions to improve gross motor skills and support transition to real-world sports participation for children with cerebral palsy. At present, this population demonstrates decreased participation in physical activities and sport compared to peers due to barriers ranging from body function to accessibility challenges. Sport provides culturally relevant opportunities for social integration, community participation and physical activity and has been shown to improve the fitness, self-esteem, confidence and quality of life of children with disabilities. The *Sports Stars* physiotherapy group has been designed to support the development of a range of fundamental movement and sports skills through activity skill practice and participation in modified popular Australian sports.

**Methods:**

This randomised, waitlist controlled, assessor blinded, superiority trial with two parallel groups will aim to compare the effectiveness of *Sports Stars* to standard care across all ICF domains. Children in the *Sports Stars* group are expected to demonstrate greater improvement in their individually-selected, sports related goals measured by the Canadian Occupational Performance Measure. This study will aim to assess sixty ambulant children aged six to 12 years with a diagnosis of cerebral palsy. Children will be excluded if they have had recent Botox or neurological/orthopaedic surgery. The *Sports Stars* intervention includes eight, one-hour, weekly physiotherapy group sessions with four to six participants and one lead physiotherapist. Outcome measures will be collected pre, post and 12 weeks post the immediate *Sports Stars* group to assess change immediately after, and at follow up time points*.*

**Discussion:**

This will be the first study of its kind to investigate a culturally relevant sports-focussed fundamental movement skills physiotherapy group for ambulant children with cerebral palsy. The findings will add to a growing pool of evidence supporting group physiotherapy for children with cerebral palsy and the *Sports Stars* group will provide an avenue for children to transition from individual physiotherapy to mainstream and modified recreational and competitive sports.

**Trial registration:**

Australian New Zealand Clinical Trials Registry: ACTRN12617000313336 Registered 28, February 2017.

WHO Universal Trial Number: U1111–1189-3355 Registered 1, November 2016.

**Electronic supplementary material:**

The online version of this article (10.1186/s12887-018-1190-z) contains supplementary material, which is available to authorized users.

## Background

Cerebral palsy (CP) is defined as a “*group of permanent disorders of the development of movement and posture, causing activity limitations that are attributed to non-progressive disturbances that occurred in the developing fetal or infant brain*” [1]. Children with CP demonstrate limitations across all International Classification of Functioning, Disability and Health (ICF) domains. They frequently exhibit body function challenges including spasticity, weakness, tightness and poor motor control leading to limitations in balance, coordination and fitness [2]. Around 59.5% of Australian children with CP are classified as level I-II on the Gross Motor Function Classification System- Expanded and Revised (GMFCS- E&R) [3]. Although these children walk without aids, they experience limitations in gross motor function, particularly in complex locomotor or object control activity skills. Children with ambulatory CP frequently fail to meet minimum physical activity guidelines [4, 5], and are even less active than their typically developing peers, with decreased frequency of participation in a more limited number of mainstream physical leisure, self-care and productivity activities, including sport [6–11].

Sport is recognised as an important part of Australian culture and is a common avenue for children to increase their physical activity [12]. For the purpose of this study, sport is defined as “*A human activity involving physical exertion and skill as the primary focus of the activity, with elements of competition where rules and patterns of behaviour governing the activity exist formally through organisations and is generally recognised as a sport* [13]*.”* Sports participation provides opportunities for social integration, community participation and physical activity and has been shown to improve the fitness, self-esteem, confidence and quality of life of children with disabilities [14, 15]. The cultural importance of sport for Australian children means that it is of even greater importance for children with disabilities to have equitable opportunities to develop gross motor function through participation in sport. Carlon [4] suggests that maintaining changes to health-related fitness requires improved physical activity behaviours in the home, school and community. Sport is one avenue to achieve this, and sports participation has been considered an alternative to prolonged physiotherapy intervention in adolescence and into adulthood [16]. By their nature, sport and physical activity interventions are more likely to occur in group formats, and children with CP who participate in group interventions have demonstrated increased engagement, motivation and participation compared to individual interventions [17–20] along with high levels of translation to real-world sports participation [21, 22]. Additionally, compared to individual physiotherapy, group physiotherapy can be more cost effective in providing the same therapy dose [23, 24].

**Fig. 1:**
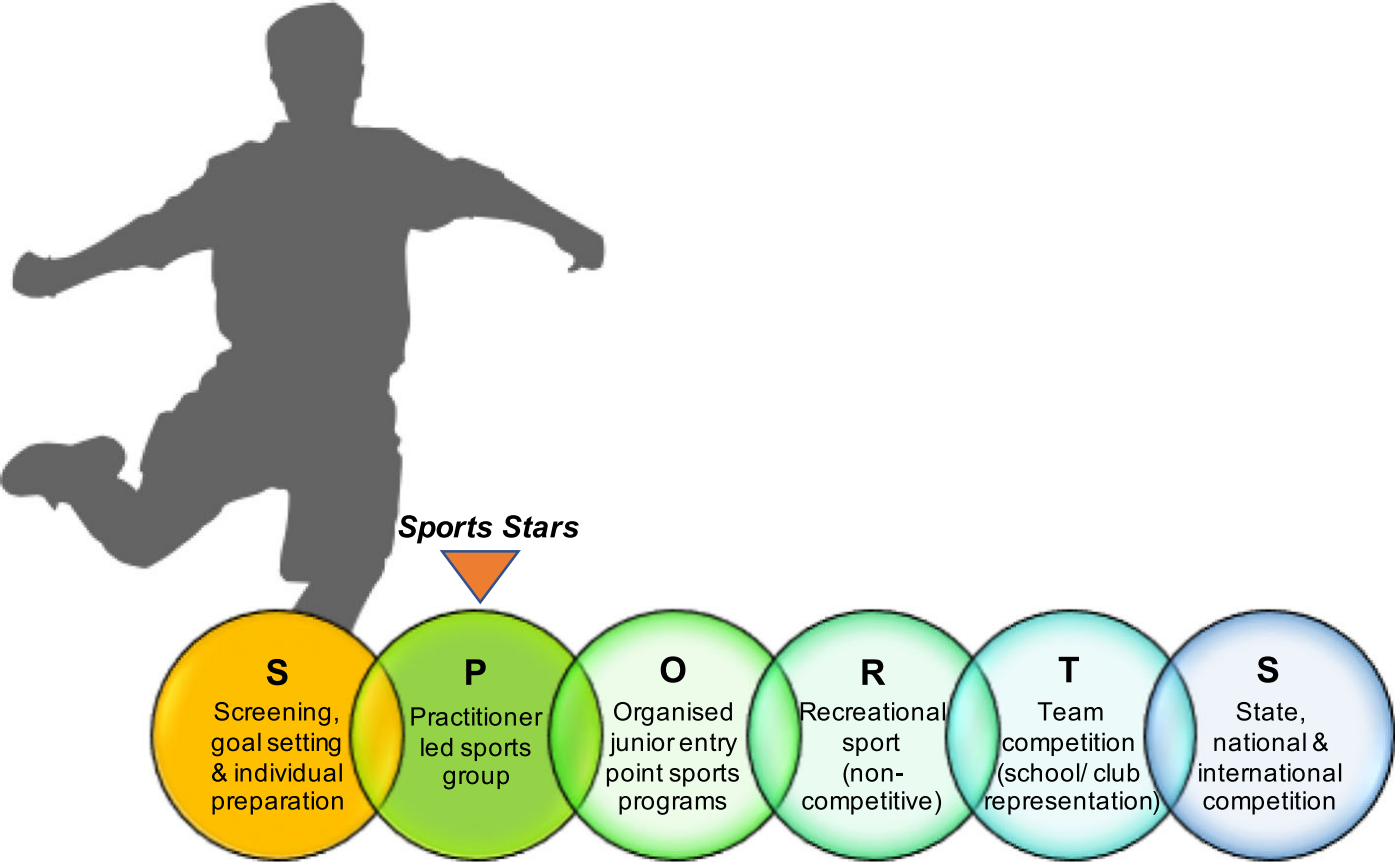
SPORTS Participation Framework for children with disabilities

Although participation of children with CP in sports has increased [25], numerous barriers to participation persist, including children’s physical ability and fatigue, accessibility of appropriate sporting opportunities and facilities, and acceptance of the child’s disability [26, 27]. Decreased experience and proficiency in fundamental movement skills is a particularly strong barrier to children’s ability to perform, and subsequently participate in, physical activity [28] as well as adversely affecting lifelong physical activity patterns [4, 29]. The SPORTS Participation Framework developed by the authors (Fig. 1) proposes a pathway for children to participate in recreational, competitive and elite level sport. Despite the evidence confirming barriers to participating in sport, there are limited opportunities for children with CP to transition from individual physiotherapy, to participating in recreational or competitive sport. A recent systematic review by our group investigating active exercise interventions targeting gross motor function in school-aged, ambulant and semi ambulant children with CP, identified Modified Sports as a promising intervention requiring further high-level research [30].

There is currently limited availability of, or evidence for, culturally relevant modified sport interventions for children with CP, with two of three low-level Modified Sport interventions identified investigating winter sports irrelevant to the Australian climate [22, 31]. *Sports Stars* targets the development of a range of fundamental movement and sports skills through activity skill practice and participation in modified popular Australian sports; soccer, netball, T-ball and cricket. This randomised controlled trial of the *Sports Stars* program will aim to fill this gap in the literature by investigating the effect of a culturally relevant, sports-oriented, group physiotherapy intervention on sports related body function, activity and participation.

## Methods/Design

### Study aims

The proposed study is a randomised, waitlist controlled, assessor blinded, superiority trial with two parallel groups. This study will aim to compare the effectiveness of a group-based, sports oriented physiotherapy intervention, *Sports Stars,* for ambulant school-aged children with CP, to standard care across all ICF domains. This study will also aim to gain feedback from treating physiotherapists and caregivers involved in the study to determine the acceptability of the intervention. The specific hypotheses to be tested are:

### Primary hypothesis

**H1:** Compared to children in the standard care group, children in the *Sports Stars* group will demonstrate greater improvement in their individually-selected, sports activity and participation related goals measured by the Canadian Occupational Performance Measure (COPM).

### Secondary hypotheses

**H2: Body function and structure:** Compared to children in the standard care group, children in the *Sports Stars* group will demonstrate greater improvements in lower limb and upper limb strength, balance, agility and their aerobic and anaerobic fitness.

**H3: Activity:** Compared to children in the standard care group, children in the *Sports Stars* group will demonstrate greater improvements in gross motor capacity, including locomotor ability and object control skills.

**H4: Participation:** Compared to children in the standard care group, children in the *Sports Stars* group will demonstrate increased participation in physical activities including recreational or formal sporting activities.

**H5: Quality of life:** Compared to children in the standard care group, children in the *Sports Stars* group will demonstrate greater improvements in quality of life as measured by parent report.

### Study sample and recruitment

#### Inclusion criteria

This study will include children who:

1. Are aged 6–12 years at study entry;
2. Have a confirmed diagnosis of CP;
3. Are ambulant without aids (classified as GMFCS- E&R Level I or II);
4. Can commit to eight, one-hour weekly group physiotherapy sessions and three, two-hour assessment appointments over a period of 6 months.

#### Exclusion criteria

Children will be excluded from the study if they:

1. Are unable to complete baseline assessments;
2. Have had orthopaedic or neurological surgery within 6 months prior to the immediate intervention start date;
3. Have had Botulinum Toxin injections within 3 months prior to the immediate intervention start date;
4. Have intellectual or behavioural difficulties which would limit their ability to participate in the assessment or therapy protocols;
5. Have medical co-morbidities which prevent them from exercising safely (e.g. cardiac or respiratory instability, uncontrolled seizures).

### Criteria for withdrawal

Participants will be excluded from the study if they fail to attend either their baseline assessment or withdraw prior to the commencement of the immediate *Sports Stars* group. Primary analysis will use the intention to treat principle, using the last observation carried forward for participants who withdraw after commencement of intervention in the immediate *Sports Stars* group.

### Recruitment

#### Sample size

According to CONSORT guidelines, sample size calculations are based on adequate power for comparison between the effects of the *Sports Stars* program compared to standard care using the COPM immediately post intervention (T2). Data from a previous study investigating the effects of an exercise group aiming to improve physical activity, balance and strength in ambulant children with CP (6–14 years) showed a standard deviation of 1.87/2.88 (intervention/control) [32]. This standard deviation and a mean change of 2 points for performance on the COPM (clinically meaningful difference) were used to calculate sample size. Based on significance (alpha) of 0.05 and 80% power, a minimum sample of 25 participants in each group (50 participants total) will be required. Therefore, 60 participants (30 in each group) will be recruited to allow for 20% attrition.

#### Recruitment process

Eligible children will be prospectively recruited through the client database of a state-wide community rehabilitation service. As geographical location and participant availability are critical to forming groups of sufficient participant numbers for this study, participants will be recruited in blocks according to their geographical location. Once at least four, and a maximum of six, children are identified that can attend an eight-week group on a specified day in a specific geographical location, these children will be assigned as group one. When a second group of four to six children are identified for a geographical location, they will be assigned as group two. When two groups of four to six participants each are identified, they will proceed to randomisation.

#### Randomisation

A random sequence will be generated via coin flip by an independent, off-site co-investigator (MA) who will not be involved with assessment or treatment (as per process used in previous studies [17]). The outcome (heads: group 1 = immediate *Sports Stars* intervention, group 2 = waitlist *Sports Stars* intervention. Tails: group 1 = waitlist *Sports Stars* intervention, group 2 = immediate *Sports Stars* intervention) will be written on a piece of paper and concealed inside a sequentially numbered, opaque envelope and stored securely off-site.

When two groups are identified, they will be randomly assigned to either the immediate intervention group or the waitlist control group with a 1:1 block allocation via drawing of one of the opaque envelopes. Randomisation will be completed offsite by the same independent co-investigator (MA). This process will continue until 60 participants complete baseline assessments and proceed to the *Sports Stars* group.

### Therapy protocols and delivery

**Fig. 2:**
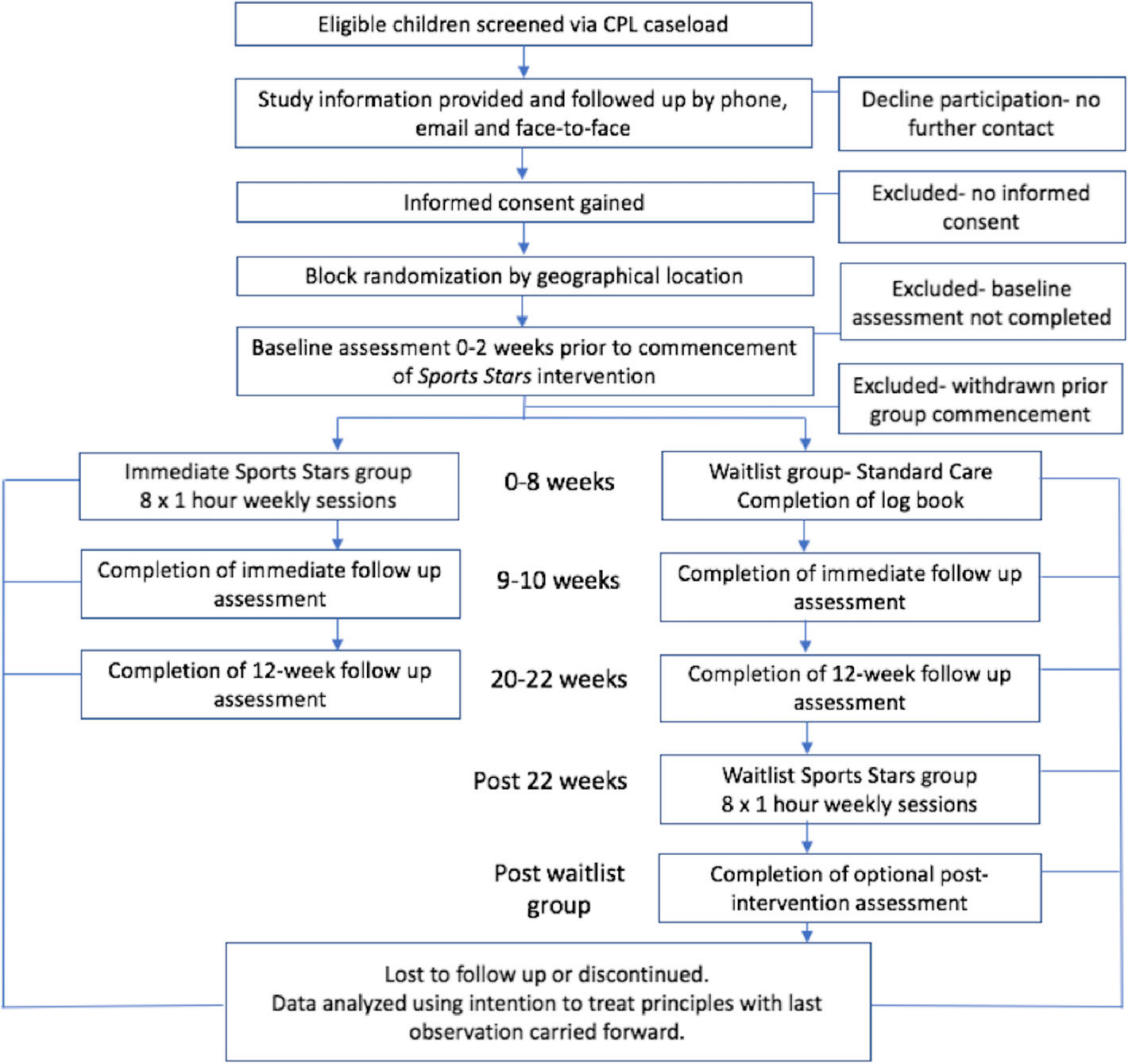
Sports Stars flow chart according to CONSORT guidelines

Refer to Fig. 2 for the study flow diagram according to CONSORT guidelines.

#### Sports Stars intervention

**Fig. 3:**
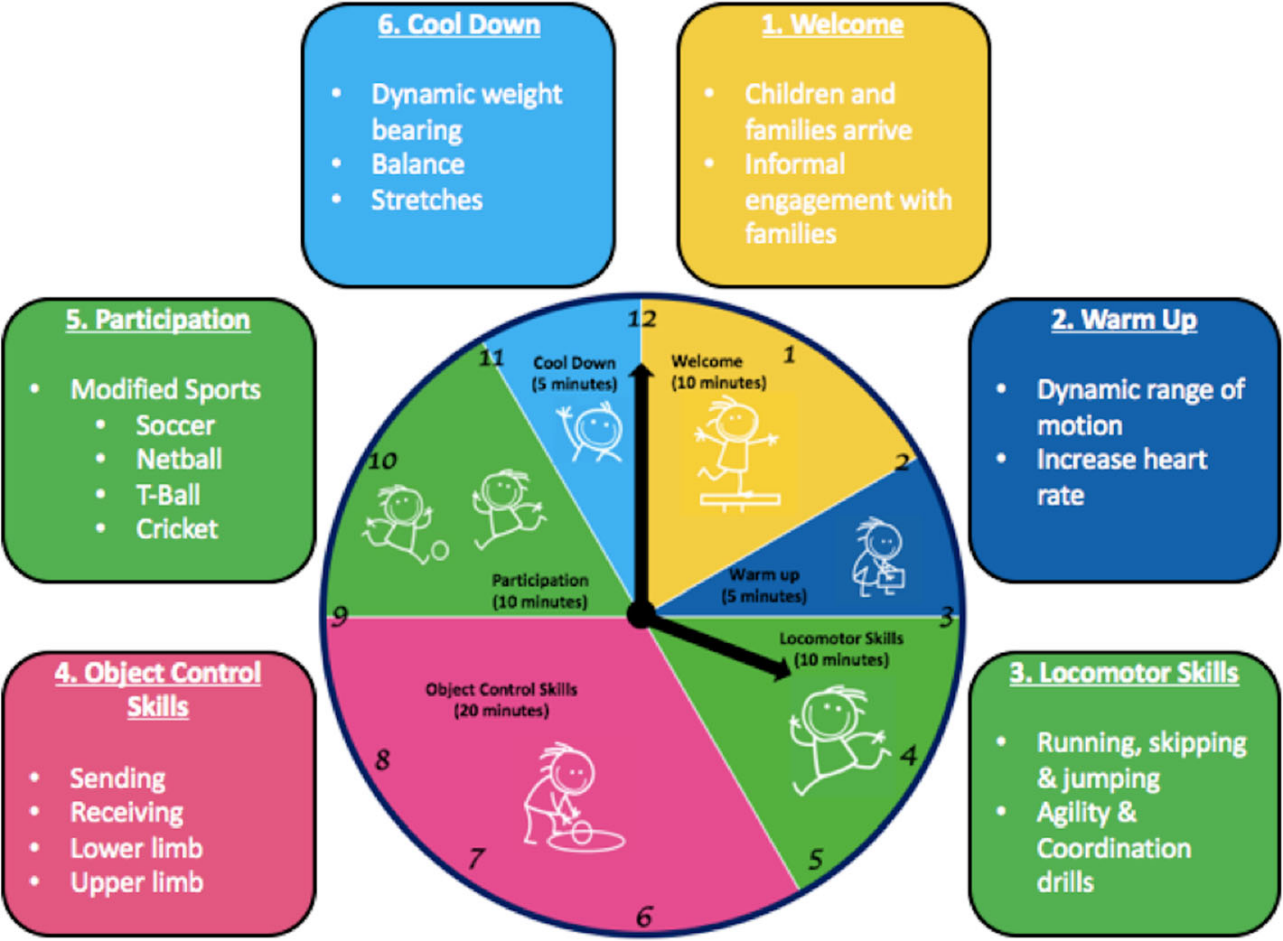
Sports Stars session content summary

Each group will contain between four and six participants with one lead Physiotherapist. The immediate *Sports Stars* group will receive eight, one-hour, weekly sessions (8 hours) of group-based, sports specific fundamental movement skills training, detailed in Fig. 3. These groups will introduce children to four popular Australian sports and support the development of core motor skills for transition to recreational mainstream and modified sports opportunities including *Junior Entry Point* sports programs, NetSetGo (netball), In2Cricket, MiniRoos (soccer) or T-Ball (softball/baseball). To achieve this, *Sports Stars* will focus on developing key *Body Functions* (aerobic and anaerobic fitness, muscle strength, balance and agility, and locomotor and object control) and sport-specific *Activity* skills to facilitate participation in modified sport games of soccer, netball, T-ball and cricket.

#### Location

*Sports Stars* will be conducted at local parks in the community in association with Queensland’s largest state-wide community physiotherapy service provider for children with CP. Groups will be provided in urban/regional Queensland, Australia, capturing the breadth of cultural and socioeconomic diversity. Each group will include back-up undercover areas to be used in the event of wet weather.

Participants of the *Sports Stars* group will not be permitted to receive other physiotherapy during their 8 week *Sports Stars* group. Throughout the duration of the study, all participants will be permitted to access standard care from other allied health (e.g. occupational therapy, speech and language pathology) and/or other adjunct therapies. Parents/guardians will be requested to record the frequency, duration and content of any standard care received including any home exercise performed.

#### Standard care comparison

Participants in the *Sports Stars* Waitlist group will receive standard care. This describes the typical intervention that a six to 12-year-old child with a diagnosis of CP would receive from their normal therapists in community, private or hospital contexts. Therapists who provided standard care will have varying degrees of experience working with children with CP. Standard care may include neurodevelopmental therapy, context-focussed therapy, strength or fitness training, or functional training. At this age, children are typically seen for 1:1 therapy, however some group therapy may also be offered. Ambulant children of this age (classified as GMFCS- E&R I-II) with CP would typically receive only 1–2 physiotherapy sessions per quarter. This may include a home program and is expected to vary between participants, however most children would receive no more than two sessions during the comparative 8 weeks of a *Sports Stars* program. Details of frequency, duration and content of any therapy session, particularly physiotherapy, will be collected via a parent log book throughout the project.

### Treatment fidelity

Intervention therapists will be masked to baseline outcome assessments. All intervention therapists providing the *Sports Stars* intervention will be qualified physiotherapists with experience working with children with CP in a community setting. At therapist training, the detailed *Sports Stars* protocol and a sports equipment pack will be provided to all therapists to ensure that participants in all groups received consistent delivery. This includes a written week by week program of sports focussed exercises and progressions (Additional file 1) with detailed instructions and video resources that therapists can use to select predetermined difficulty levels to suit each child’s age, goals and preferences. Therapists will receive individual, face to face training in the *Sports Stars* protocol by the first author prior to the commencement of the group. Treating therapists will engage in further consultation as required with the study coordinator either by phone or in person to review the *Sports Stars* package and to discuss program content and structure prior to providing the group to participants. Each child will have features of their participation in each group session recorded by the treating therapist, including exercise type, level of difficulty and therapist observed engagement. At week three and six of the study, each therapist will undergo fidelity review and receive guidance via email through an independent, offsite co-investigator (MA) who will not be involved with assessment or treatment regarding intervention format, content, progression and data recording. Independent content analysis will determine compliance with the provided protocol. Parents of both the immediate and waitlist groups will be provided with training journals to record any therapy, including home exercises, and sport that they undertake during the study period.

### Outcome measures and procedures

All outcomes will be collected pre-intervention, immediately post intervention and at 12 weeks post intervention by the blinded chief investigator (GC).

1. Classification of Sample

Participants will be classified based on their severity and age to compare groups at baseline. They will be classified according to:

- **Gross motor function:** *Gross Motor Functional Classification System (GMFCS-E&R)* [33]

The Gross Motor Function Classification System is an internationally recognized classification system for children and youth with CP. Participants will be classified as either classified as GMFCS- E&R I (able to walk independently at home, school, outdoors and in the community with limitations in speed, balance or coordination) or II (able to walk in most settings, however may have difficulty with distances or more challenging environments and gross motor skills like running and jumping) using the descriptors for between the child’s 6th and 12th birthday [33].

b. **Classification of CP**

Participants will be classified by motor type (spasticity, dyskinesia, ataxia or unclassifiable) and distribution (unilateral or bilateral) [34, 35].

c. **Functional mobility:** *Functional Mobility Scale (FMS)* [36]

Participants will be classified by their ability to walk five, 50 and 500 m, correlating to their ability at home, school and community. Scores range from N (does not apply), to 6 (independent on all surfaces without aids) [36].

2. **Outcomes:** Measured for all participants at four time points and compared to baseline and each other:

- Time one (T1): *zero months*- Baseline
- Time two (T2): *2 months*- Immediately after immediate intervention
- Time three (T3): *5 months*- 12 weeks after immediate intervention

For children participating in the waitlist group, an optional fourth assessment will occur:

- Time four (T4): approximately *8 months*- Immediately after waitlist intervention

### Body function and structure outcomes

#### Aerobic capacity & agility: *10 × 5 Meter Sprint Test* [37]

The 10 × 5 Meter Sprint Test is designed to measure aerobic capacity and agility in children with CP of GMFCS- E&R level I or II. Children must continuously sprint the five-meter course 10 times, making turns at the cones marking the end of the five meters. The 10 × 5 Meter Sprint Test has excellent inter-observer (ICC > 0.97) and test-retest reliability (*r* = 1). It has reported good construct validity. The 10 × 5-m sprint test is sensitive to change for children at GMFCS- E&R levels I and II and therapists report a high clinical feasibility. A decrease in exercise time of 3.2 s would be considered real change [37].

#### Anaerobic Capacity *Muscle Power Sprint Test (MPST)* [37]

The MPST measures anaerobic capacity by asking the participant to sprint 15 m (marked by lines and cones) at their maximum pace, 6 times, with 10 s recovery between each sprint. The MPST has a high inter-observer and test-retest reliability (*r* = 0.97–0.99). It has good construct validity with GMFCS- E&R [37] and the Wingate Anaerobic Test (Peak Power: *r* = 0.731, Mean Power: *r* = 0.903) [38]. It is sensitive to change in children GMFCS- E&R level I and II and has high clinical feasibility. Standard errors of measurement were reported at 13.9 (peak power) and 9 (mean power) Watts [37].

#### Functional Lower Limb Strength: *Standing Broad Jump* [39]

The standing broad jump measures lower limb strength in the context of sports participation. Standing with toes up to a line, children are asked to jump forward as far as they can, landing with both feet. The distance between the start line and the most distal part of their toes of their back foot will be measured for three jumps, with the average recorded to the nearest centimetre. The standing broad jump has excellent test-retest reliability in typically developing children (ICC- 0.88 [40]) and in children with down syndrome (ICC- 0.89 [41]). It has excellent concurrent validity with measures of physical fitness (*r* = 0.84) [40], Paralympic throwing (*r* = 0.77–0.86) [42] and sprinting (*r* = 0.82) [43] for children with disabilities. It has been reported to be sensitive to change as part of a test battery and is a feasible clinical test [40].

#### Functional Lower Limb Strength: *Vertical Jump* [39]

The vertical jump measures lower limb strength. Standing next to a wall, children raise their arm. The most distal point of their fingers is marked. They are instructed to jump as high as they can, a second mark being made at the height of their jump. The vertical distance of three jumps will be measured and averaged to obtain the jump height to the nearest centimetre. The vertical jump has been used to represent the core functional output of children’s strength in previous literature [44]. It is also utilised readily in mainstream sport and physical education and is included in Australia’s national talent identification and development program [39].

#### Functional Upper Limb Strength*: Seated throw* [39]

The seated throw measures functional upper limb strength. Children are seated comfortably with their back against a wall. Using a chest pass, they are asked to throw a basketball as far as possible while keeping their back against the wall. The distance between the wall and the first point of contact of the ball will be measured to the nearest centimetre for three throws and the average calculated. Similar to the vertical jump, the seated throw has been used to represent the core functional output of children’s strength in previous literature [44, 45], is common and clinically feasible in mainstream sport and physical education and is included in Australia’s national talent identification and development program [39].

### Activity and Participation outcomes

#### Individual activity and participation based goals: *Canadian Occupational Performance Measure (COPM)* [46]

The COPM is the most frequently used measure of individual client centred outcomes in paediatric rehabilitation [47]. It measures individual, client-centred outcomes by focussing on the goals and priorities of the child and family [48]. The child-adapted model of the COPM will be administered via semi-structured interview with the parent/caregiver and child. Three sports related goals (at least one activity and one participation focussed) will be identified by caregivers. Ratings scale of their child’s performance and their satisfaction with this performance will be made on a 1–10 ordinal scale. The COPM has high re-test reliability (ICC 0.76–0.89). It demonstrates concurrent validity with the Functional Independence Measure and Klein-Bell [49] in addition to construct and criterion validity [50]. It has good sensitivity to change [49]. On the ordinal scale (1–10) a change of two or more points is considered clinically meaningful [51].

#### Functional Mobility and Balance: Timed up and go (TUG) [52]

The TUG is a simple measure of balance, anticipatory postural control and functional mobility. The modified procedure for children described by Williams et al. [52] requires participants to stand up from a chair with a backrest but no arms, walk three meters to touch a target before turning and returning to a seated position. They are timed from their bottom rising from the seat to touching back down on the seat and are given encouragement throughout the procedure. The TUG has a high within-session and test-retest reliability (ICC 0.99) [53]. It is reported to be an ecologically valid tool. The TUG is responsive to change over time in children with physical disabilities [52]. Minimal detectable changes of 1.4 s (GMFCS- E&R I) and 2.87 s (GMFCS- E&R II) have been calculated [54].

#### Gross Motor Capacity (CP Specific): Gross Motor Function Measure Challenge Module (GMFM Challenge) [55]

The GMFM Challenge was developed as an extension of the GMFM which is used internationally to quantify gross motor performance in children with CP. The GMFM can have a ceiling effect, especially for children of GMFCS- E&R I classification over the age of 5 years. The GMFM Challenge is an observational measure of high-level skills, speed and quality of performance in children with ambulatory CP. It has been found to have excellent inter-rater (ICC = 0.97) and test-retest reliability (ICC = 0.96) [56]. The content validity of the GMFM Challenge was enhanced by using existing, feasible and relevant observational gross motor measures, working with experienced clinicians and performing participant based content validity checking with children with CP. Rasch analysis has been completed but not yet published [57]. Preliminary minimal detectable change values have been reported at 7.17–8.44 [56].

#### Gross Motor Capacity (Sport Specific): *Test of Gross Motor Development-2 (TGMD-2)* [58]

The TGMD-2 is an observational measure of gross motor skill performance relating to sports. It assesses skills in two categories, locomotor and object control, each with six items. In doing this, the TGMD-2 focusses on specific sports skills and is often used for children in mainstream education or sporting contexts and has been reported to be the gold standard for gross motor skill for pre-schoolers [59]. It is reported to have high inter-rater, test-retest, internal and composite reliability [58, 60–62]. Ulrich [58] reports excellent validity and clinical feasibility in the TGMD-2 manual and studies have demonstrated construct validity [61] and concurrent validity with the GMFCS- E&R [62], Pre-schooler Gross Motor Quality Scale [59] and measures of physical fitness [63].

#### Participation Frequency and Enjoyment: *Children’s Assessment of Participation and Enjoyment (CAPE) and Preferences of Activities for Children (PAC)* [64]

The CAPE and PAC are questionnaires that measure participation of children in a range of activities outside of school. The CAPE-PAC measures who a child is participating with, enjoyment of an activity and the diversity and intensity of participation in formal (organised sport, other skill-based activities, and clubs, groups and organisations) and informal activities (recreational, active-physical, social, skill-based, and self-improvement). It has adequate test-retest reliability (ICC = 0.67–0.86) [65]. There is evidence for construct and face validity and clinical utility [65, 66].

### Contextual

#### Quality of Life: *Cerebral Palsy Quality of Life- children’s version (CP QOL-Child)* [67]

The CP QOL-Child is a quality of life questionnaire that assesses wellbeing in seven domains. It was specifically designed for children with CP aged 4–12 years. Parent proxy reports will be used in this study due to the age of most children anticipated in the *Sports Stars* group. The CP QOL-Child demonstrates high internal consistency (ICC 0.74–0.92) and test-retest reliability (ICC 0.76–0.89) for the parent proxy report. It demonstrates adequate construct validity relative construct validity with the Child Health Questionnaire, KIDSCREEN and GMFCS- E&R [67].

#### Caregiver satisfaction

Post intervention (T2) a custom-designed questionnaire will be used to measure satisfaction with the *Sports Stars* program. Questions will relate to group design, group content, and satisfaction with sport readiness. Outcomes will be measured on a *Sports Stars* specific eleven point Likert Scales with an additional open-ended question in each category. Answers will be reviewed by the primary investigator (GC) to determine consistent themes.

#### Child’s motivation and engagement within the *Sports Stars* group

To evaluate if there is a relationship between study outcomes and participant engagement, treating physiotherapists will be asked to record participants’ engagement in each component of the group intervention (warm-up, locomotor skills, object control skills, game participation and cool-down), in each therapy session using *Sports Stars* specific five point Likert Scales. Thematic analysis will be performed by to determine overarching themes.

## Adverse events

Adverse events will be reported by the treating Physiotherapist as per organisational policy. Standard organisational response and follow up will occur based on the severity of the adverse event. Any reported adverse events will be recorded by the treating physiotherapist following each session and provided to investigators at the completion of the group. They will be classified as Insignificant: no discernible injury, Minor: first aid treatment required, Moderate: medical treatment required, Major: extensive injury, or Catastrophic: resulting in death or persistent disability.

## Analyses

Statistical analysis will be performed using SPSS statistical software. Primary analysis will use the intention to treat principle, using the last observation recorded for participants who withdraw from the program.

Baseline data will be reported using descriptive statistics for each variable (individual sports related activity and participation goals, GM capacity, aerobic fitness, anaerobic fitness and agility, functional strength, participation and quality of life) to establish any difference between randomised groups. The method of aggregation will depend on the normality of the data and will include the mean and standard deviation (normally distributed data) or the median and interquartile range (non-normally distributed data). If characteristics are not comparable at baseline, they will be modelled as covariates in subsequent analyses in order to adjust their possible confounding effects. Linear mixed models will be used to evaluate the effectiveness of the *Sports Stars* intervention compared to the waitlist control on the primary outcome (COPM). Linear mixed models take into account variation in individuals over time, are able to manage missing data without excluding participants for further analysis and examine changes in the outcomes over time as well as across the two groups. Significance will be set at *p* < 0.05. Residuals of the fitted models will be examined to ensure that all required assumptions are met.

## Discussion

This protocol paper presents the background and design of a randomised controlled trial designed to investigate the effectiveness of delivering sports-focussed fundamental motor skills therapy, *Sports Stars,* for children with CP through a group-based service model compared to standard care. To our knowledge this will be the first study of its kind to investigate a culturally relevant sports-based physiotherapy group in this population in Australia. It will add to a growing body of evidence supporting group therapy for children with CP and provide an avenue for children to transition from individual therapy to participating in junior entry-level sport programs such as MiniRoos, NetSetGo, T-Ball and in2Cricket, and onto recreational and competitive mainstream and modified sport. This study will encourage therapy providers to engage with flexible service delivery in response to client and family preferences and goals, and inform parents and carers in deciding how to allocate their funding.

## Additional file

Additional file 1: Sports Stars sample session plan (PDF 41 kb)

## Abbreviations

CAPE-PAC: Children’s assessment of participation and enjoyment and preferences of activities for children
COPM: Canadian occupational performance measure
CP QoL-child: Cerebral palsy quality of life- children’s version.
CP: Cerebral palsy
CPL: The Cerebral Palsy League of Queensland
FMS: Functional mobility scale
GC: Georgina Clutterbuck
GMFCS- E&R: Gross motor function classification system- extended and revised
GMFM Challenge: Gross motor function measure- challenge module)
ICC: Intraclass correlation coefficient
ICF: International classification of functioning, disability and health (ICF)
LJ: Leanne Johnston
MA: Megan Auld
MPST: Muscle power sprint test
T1: Time one
T2: Time two
T3: Time three
T4: Time four
TGMD-2: Test of gross motor development- version two
TUG: Timed up and go
