# Supplementary material for: SPORTS STARS study protocol: a randomised, controlled trial of the effectiveness of a physiotherapist-led modified sport intervention for ambulant school-aged children with cerebral palsy
Source: BMC Pediatr. 2018 Aug 2;18:258. doi: 10.1186/s12887-018-1190-z (PMC6090768; doi:10.1186/s12887-018-1190-z)
Supplement: Supplementary file 1 — Sports Stars sample session plan (PDF 41 kb) [file 12887_2018_1190_MOESM1_ESM.pdf]

Program Name: Sports Stars **Facilitators:** ______________________
Child's Name: ______________________ **Attending with:** ______________________
Week Number: 1 Soccer **Date:** _____/_____/__________

Activity	Level 1	Level 2	Level 3	Notes
**Warm Up (5 min) "Let's get our bodies sports ready!"**				**Engagement:** 1 2 3 4
**Range of motion**<br>***Upper limb / trunk***<br>• Roll shoulders<br>• Circle arms<br>***Lower limb / trunk***<br>• Standing toe touch<br>• Stork stand (quad stretch)<br>• Ankle circles (SLS +/- hold for balance)	**Increase heart rate**<br>• High knees<br>• Jump up/down; left/right<br>• Modified burpees			
**ACTIVITY- Locomotor Skills (10 min) "How fast are your feet?"**				**Engagement:** 1 2 3 4
**Running**<br>• Straight relay race	Stops to pass, slow	Slows down to pass, faster	Racing swiftly with smooth pass	
**Agility & Coordination**<br>• Weaving	Large arc around cones	Smaller arc, more forward facing body.	Small arc close to cones. Timed to increase speed	
• Toe tapping on soccer ball	Slowly, one foot at a time	Increase speed	Hopping- moments of no ground contact	
• Colour Square	Large delay direction change.	Small delay in direction change, faster	Swift & coordinated direction change.	
**ACTIVITY – Object Control Skills (20 min) "Let's have a ball!"**				**Engagement:** 1 2 3 4
**Eye-Foot Coordination**<br>Stationary kicking into a goal	Goal 1.5m away. Kicks without stepping	Goal 3m away. Steps beside ball - kick with other foot.	Goal 5m away. Runs to ball and kicks with appropriate force.	
Dribbling drills<br>- To far marker and back in straight line<br>- Weaving around markers in a zig zag pattern.	Walking with large arc around cones.	Slow run with a wide arc around the far cone.	Running with smaller arc around the far cone. Timed/ competitive	
**Eye-Head Coordination**<br>Goal protection- Heading balls	Large ball thrown directly to child's head	Medium ball to either side requiring step / lean.	Soccer ball thrown to either side or ahead- must step.	
**PARTICIPATION - Modified Sports Games (10 min) "It's game time!"**				**Engagement:** 1 2 3 4
**Modified Games**<br>**Week 1 & 2: Soccer**<br>Modify as necessary.	All participants work on the same team.	Children work on different teams.	Children work on different teams, include goal protection.	
**COOL DOWN (5 min) "2Cool4School"**				**Engagement:** 1 2 3 4
**Cool down**<br>"Animal Yoga" – Dynamic weight bearing stretches, balance and cool down<br>- Downward dog (Hamstring and calf stretch)<br>- Happy cat, angry cat (trunk ROM)<br>- Flamingo (SLS)<br>- Giraffe (side lunge- adductor & lateral trunk stretch)<br>- Butterfly (short adductor stretch)				
Notes				

AHP Name:______________________________ **Signature:**______________________________
